# Development and usability evaluation of a patient journey-based mHealth intervention for esophageal cancer patients: a three-phase protocol

**DOI:** 10.3389/fpubh.2025.1717833

**Published:** 2026-01-09

**Authors:** Danyu Ji, Li Yang, Xinyue Jiang, Jiang Fu, Tingting Dai, Li Yu, Haining Zhou

**Affiliations:** 1 Department of Thoracic Surgery, Suining Central Hospital, Suining, China; 2 School of Nursing, Zunyi Medical University, Zunyi, China; 3 Department of Nursing, Nanchong Central Hospital, Nanchong, China; 4 Department of Physical Examination, Suining Central Hospital, Suining, China

**Keywords:** esophageal cancer, digital health, mHealth, WeChat mini-program, patient journey, patient-centered care, usability evaluation, protocols

## Abstract

**Introduction:**

Esophageal cancer (EC) patients face significant challenges in health management across different disease stages, including symptom monitoring, treatment adherence, and long-term self-care. Digital health interventions, particularly mobile health (mHealth) applications, hold promise for providing personalized support and improving patient engagement. However, many existing mHealth tools for esophageal cancer lack a strong theoretical foundation and fail to fully incorporate patient-centered considerations, which may limit their long-term effectiveness. This study aims to develop and evaluate a WeChat-based mini-program that integrates Patient Journey Mapping (PJM) and the Integrated Theory of Health Behavior Change (ITHBC) to support personalized health management for esophageal cancer patients.

**Methods:**

This study will adopt a single-arm, exploratory design. Semi-structured interviews with esophageal cancer patients will first be conducted to identify their health management needs. Based on these findings, a WeChat-based mini-program will be developed and iteratively refined through Delphi expert consultation. In the evaluation phase, a mixed-method usability assessment will be performed, incorporating the mHealth App Usability Questionnaire (MAUQ), task-based performance metrics, and backend interaction analytics. To supplement these quantitative measures, brief post-test interviews will be conducted to capture user experiences and contextual factors. The primary outcome is system usability. Secondary outcomes include task completion performance, while backend interaction data will serve as process indicators of user engagement.

**Discussion:**

The findings of this study will provide insights into the user experience and system functionality of a WeChat-based mini-program designed for esophageal cancer patients. The results will inform refinements to the intervention, ensuring better alignment with patient needs and enhancing the feasibility of digital health solutions in oncology care. Furthermore, by integrating patient journey insights, this study helps establish a more patient-centered approach to mHealth design. This approach may serve as a foundation for future digital health tools supporting cancer care and long-term health management.

**Clinical trial registration:**

https://www.chictr.org.cn, Chinese Clinical Trial Registry: ChiCTR2500098611.

## 1 Introduction

Esophageal cancer (EC) is an aggressive malignancy and ranks as the sixth leading cause of cancer-related mortality worldwide (1). In 2020, an estimated 604,000 new cases and 544,000 deaths were reported globally (2), with China accounting for more than half of the total burden (3, 4). Despite progress in multimodal treatment, including surgery, chemotherapy, and radiotherapy (5), the overall prognosis remains poor, with a 5-year survival rate of approximately 30% (6). The disease trajectory is often complex, characterized by a high symptom burden, post-operative complications, and significant impairments in quality of life (7–9).

Effective long-term management is therefore essential for improving prognosis and quality of life (10). Patients often face multiple health challenges throughout the disease trajectory, including dysphagia, malnutrition, fatigue, and psychological distress (11–14). Although health management needs have been recognized (15, 16), most existing interventions focus on isolated symptoms, such as pain control or nutritional issues (17–19), rather than addressing the disease trajectory comprehensively. Personalized health management interventions have been shown to significantly improve symptom control and enhance patients' quality of life (20, 21). However, conventional approaches are constrained by limited healthcare resource, suboptimal patient adherence, and poor information accessibility, making continuous and individualized support difficult to implement.

With the rapid advancement of mobile internet technologies, mobile health (mHealth) interventions have become integral to chronic disease management and cancer care (22, 23). These interventions help overcome the temporal and spatial constraints of traditional healthcare models (24), delivering personalized and accessible healthcare solutions while potentially reducing medical costs (25, 26). WeChat Mini Programs, a widely adopted and cost-effective digital platform, have been increasingly utilized in health interventions, demonstrating their potential to enhance patient self-management and improve health outcomes (27, 28).

However, despite the promising applications of mHealth tools in disease management, existing digital health interventions are primarily designed based on expert opinions and literature reviews, which may fail to fully capture patients' actual needs, leading to suboptimal user engagement and low long-term adherence (29, 30). Moreover, the lack of robust theoretical frameworks and empirical validation further limits their clinical applicability, highlighting the need for more patient-centered and evidence-based approaches.

Patient Journey Mapping (PJM) is a valuable tool for systematically identifying patients' evolving needs throughout disease progression (31, 32). Addressing the limitations of expert-driven intervention design, PJM provides a structured approach to capturing real-world patient experiences, encompassing physical symptoms, psychological distress, and social challenges (33, 34). Through methods such as in-depth interviews and patient-reported outcomes, PJM uncovers care gaps and informs service optimization (35). In EC care, where patients navigate a prolonged and complex treatment journey, PJM-based mHealth strategies offer a promising framework for developing targeted, patient-centered interventions. While PJM has been successfully incorporated into mHealth design for selected chronic and population-specific conditions (36–38), its potential in EC remains unexplored. Given the multifaceted needs of EC patients, leveraging PJM as a foundation for mHealth interventions could enhance patient engagement, improve long-term adherence, and optimize health management.

As the theoretical foundation of this study, the Integrated Theory of Health Behavior Change (ITHBC) was selected to guide the intervention development. Traditional models such as the Health Belief Model (HBM) and Transtheoretical Model have been widely used in behavior change research (39, 40); however, they tend to emphasize individual cognition or discrete stages and do not provide an integrative, process-oriented perspective for sustaining long-term behavior change. In contrast, ITHBC combines multiple theoretical perspectives and emphasizes a patient-centered, interactive, and motivational process of behavior change (41). Its core constructs include self-efficacy, emotional support, and social facilitation, which collectively promote the transition from health beliefs to actions and help maintain health-promoting behaviors (42, 43). Given its comprehensive, process-based structure, ITHBC is well-suited to inform the design of personalized, stage-based mHealth interventions for EC patients.

This study adopts the patient journey approach to systematically identify key challenges and employs ITHBC as a theoretical foundation to guide the design of a WeChat-based mini program for EC management. The primary objective is to assess the usability and user experience of the platform, examining its functionality, ease of use, and relevance to patient needs. Findings from the usability evaluation will inform iterative refinements and guide future research directions.

## 2 Materials and analysis

**Figure 1:**
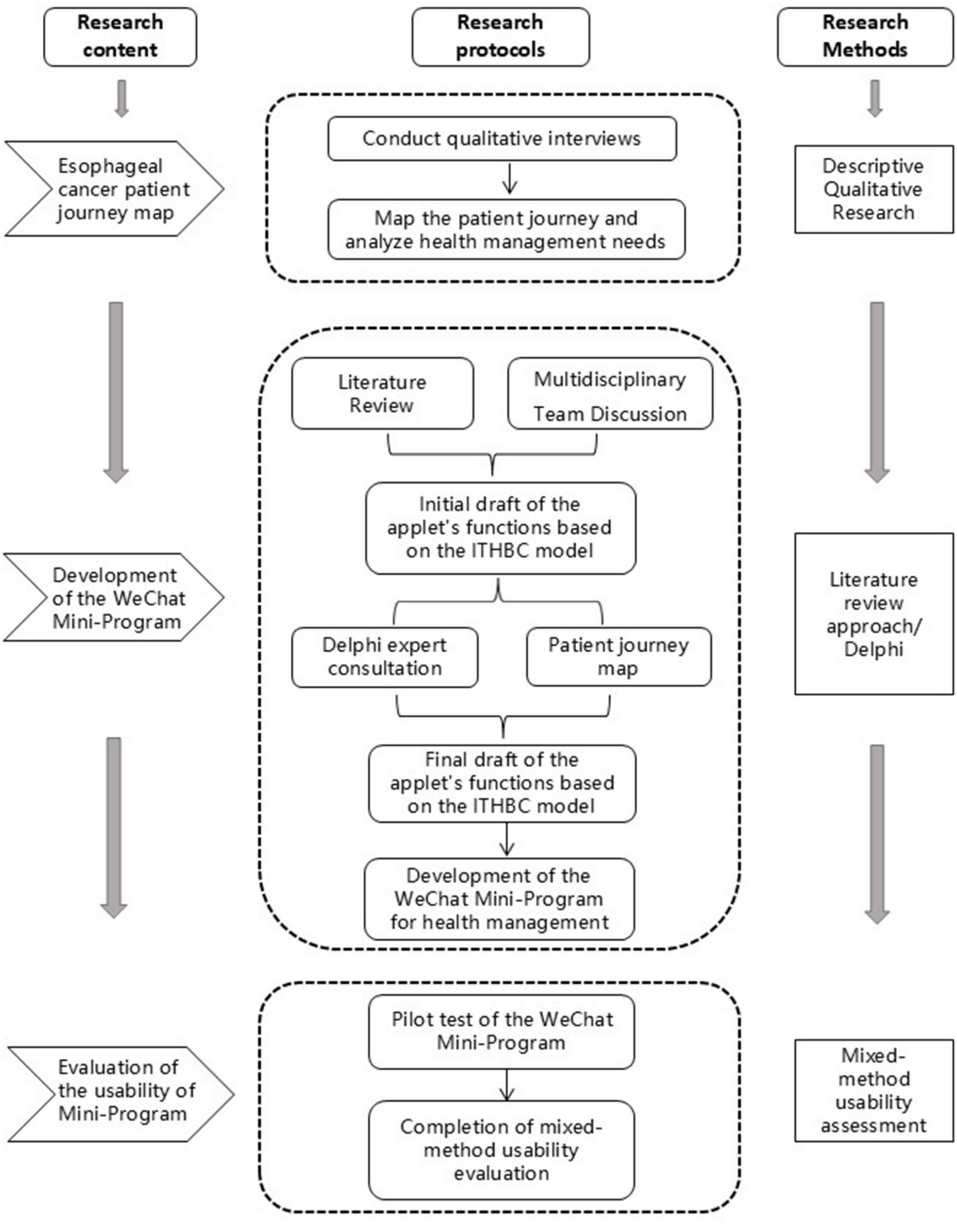
Overview of the study design and key methodological components. The figure presents the three-phase structure of the study, comprising Phase 1 (semi-structured interviews to identify patient needs), Phase 2 (mini-program development and expert refinement), and Phase 3 (mixed-method usability evaluation using quantitative metrics and post-test interviews). Key activities and expected outputs of each phase are summarized to illustrate the overall research process.

This study adopts a single-arm, exploratory design comprising three sequential phases to guide the development and preliminary evaluation of a WeChat-based digital health platform for patients with esophageal cancer (EC). Phase 1 applies Patient Journey Mapping (PJM) to systematically assess patient needs and care experiences. Phase 2 integrates patient insights and expert recommendations into the platform's design. Phase 3 evaluates the platform's usability and acceptability to ensure alignment with patient needs. The timeline of enrolment, development, and evaluation activities is detailed in Table 1, and the study design flowchart is shown in Figure 1.

**Table 1:** Study schedule for enrolment, data collection, and usability evaluation.

**Timepoint/Phase**	**Week-1 (Preparation)**	**Phase 1: Qualitative Interviews (Month 1–2)**	**Phase 2: Platform Development (Month 3–4)**	**Phase 3: Usability Testing (Month 5–7)**
Eligibility and Consent		√		√
Demographics^*^		√		√
Interview Guide Development	√			
Qualitative Interviews		√		
Delphi expert consultation			√	
Platform Development Planning	√		√	
Task-based Testing				√
mHealth App Usability Questionnaire (MAUQ) ^**^				√
Backend Data Collection				√
Post-test interviews				√

^*^Eligibility and demographics were collected separately for Phase 1 and Phase 3 participants.

^**^mHealth App Usability Questionnaire (MAUQ) was administered after participants had used the app for 1 month.

The study will conform to the principles of the Declaration of Helsinki. This study was approved by the Medical Research Ethics Committee of Suining Central Hospital (KYLLKS20250009) and is registered in Chinese Clinical Trial Registry (Registration Number: ChiCTR2500098611) before the enrollment of the first participant. In addition, this mHealth intervention will be guided by the WHO mHealth Evidence Reporting and Assessment (mERA) (44) checklist to ensure comprehensive documentation of the intervention components and implementation procedures.

### 2.1 Phase 1: patient journey mapping

The first phase, Patient Journey Mapping (PJM), aims to gain in-depth insights into the experiences and challenges of EC patients. A descriptive qualitative approach will be employed, using semi-structured interviews to explore patients' self-management challenges and support needs. Findings from this phase will inform the design of core functionalities for a patient-centered digital health platform.

#### 2.1.1 Sample/participants

Participants will be recruited from the thoracic surgery and oncology departments of a tertiary hospital in Sichuan Province. Eligible participants must have completed primary treatment, such as surgery, neoadjuvant therapy, and/or adjuvant therapy, and be within 3–12 months post-treatment. The inclusion criteria are as follows: (1) histopathologically confirmed diagnosis of esophageal cancer according to standard clinical diagnostic criteria (45); (2) age between 18 and 75 years; (3) adequate cognitive function and the ability to communicate effectively; (4) voluntary participation with signed informed consent. The exclusion criteria include: (1) concurrent diagnosis of other malignancies; (2) end-stage disease, defined as an expected survival of ≤ 6 weeks; (3) severe cardiovascular disease, or hepatic or renal failure; (4) history of psychiatric disorders or cognitive impairment.

#### 2.1.2 Sample size and recruitment

Potential participants will be identified through follow-up outpatient clinics. Researchers will collaborate with clinic physicians to screen individuals who meet the inclusion criteria. Eligible candidates will be approached during their scheduled follow-up visits and invited to participate in a retrospective semi-structured interview.

A maximum variation sampling strategy will be employed to capture diverse perspectives, ensuring variation in sociodemographic backgrounds and treatment experiences. Based on the principle of data saturation, approximately 18 participants will be recruited. Recruitment will conclude when no new themes or concepts emerge from subsequent interviews.

#### 2.1.3 Data collection and management

Semi-structured interviews will be conducted by a trained researcher. Drawing on a comprehensive literature review and expert consensus (46), a structured interview guide will be developed to explore key domains of the esophageal cancer care experience. Open-ended questions will be employed to facilitate in-depth exploration of participants' experiences. A summary of the interview domains and sample questions is provided in Table 2.

**Table 2:** Overview of semi-structured interview questions about participants' health journey.

**Topic**	**Key question**
Health Issues and Symptom Management	Initial symptom characteristics; treatment-related discomfort; post-operative challenges; persistent rehabilitation issues
Psychological and Emotional Support	Emotional reactions to diagnosis; anxiety during treatment; adequacy of support systems; demand for psychological interventions
Health Information Needs and Accessibility	Information sources; treatment comprehension; rehabilitation guidance needs; barriers to reliable information; Unmet needs
Healthcare Experience and Communication	Provider communication quality; hospital process efficiency; communication barriers; service improvement suggestions
Demand for Health Management Tools	Mini-program adoption willingness; functional requirements; content format preferences; caregiver assistance needs

Prior to the interview, the researcher will establish rapport with participants, introduce the study objectives, and obtain written informed consent. Interviews will be conducted either in a private hospital setting or via secure, encrypted online platforms, depending on participants' preferences. Each session will last approximately 40–50 min. With participants' consent, all dialogues will be audio-recorded, and non-verbal cues will be documented.

Transcriptions will be generated verbatim and anonymized to protect confidentiality. Each dataset will be assigned a unique identifier, with all personally identifiable information removed. Data will be securely stored with restricted access, available only to authorized research team members.

#### 2.1.4 Qualitative data analysis and patient journey mapping

Qualitative data will be analyzed using conventional content analysis. Audio recordings will be transcribed verbatim within 24 h of each interview to maintain contextual accuracy. Two independent researchers will code the transcripts using NVivo 12 Plus software, with discrepancies resolved through discussion or third-party review.

A patient journey map will be constructed to visually represent patients' evolving needs across distinct treatment phases. The initial framework will be established based on a literature review and clinical expertise. Interview data will be iteratively analyzed and incorporated to refine the framework, ensuring a comprehensive representation of patient experiences (47). To enhance the credibility and patient-centeredness, patient representatives will be invited to review the journey map and provide feedback (48).

### 2.2 Phase 2: development of the WeChat mini-program

This phase aims to design and develop a WeChat-based mini-program to support the health management of EC patients, based on insights from Phase 1 and refined through input from clinical experts. The platform will be developed through an iterative process incorporating feedback from patients, clinicians, and digital health experts, ensuring patient-centered design and continuous alignment with evolving clinical practices and evidence.

#### 2.2.1 Establishment of a multidisciplinary research team

(1) Team composition: a multidisciplinary research team is established, including senior nurses, attending physicians, nursing informatics specialists, dietitians, psychologists, software engineers, and graduate nursing students. This collaborative approach fosters effective interdisciplinary integration, enhancing both the scientific rigor and practical applicability of the mini-program.
(2) Team responsibilities: the team is responsible for transforming qualitative research findings into functional specifications, verifying content for medical accuracy and scientific validity, and developing the program within the WeChat ecosystem. Iterative testing is conducted to refine and optimize the mini-program.
(3) Quality control: biweekly meetings are held to monitor development progress. A structured review process ensures compliance with quality standards, and any issues encountered are promptly addressed to maintain consistency and reliability.

#### 2.2.2 App functionality modules

**Figure 2:**
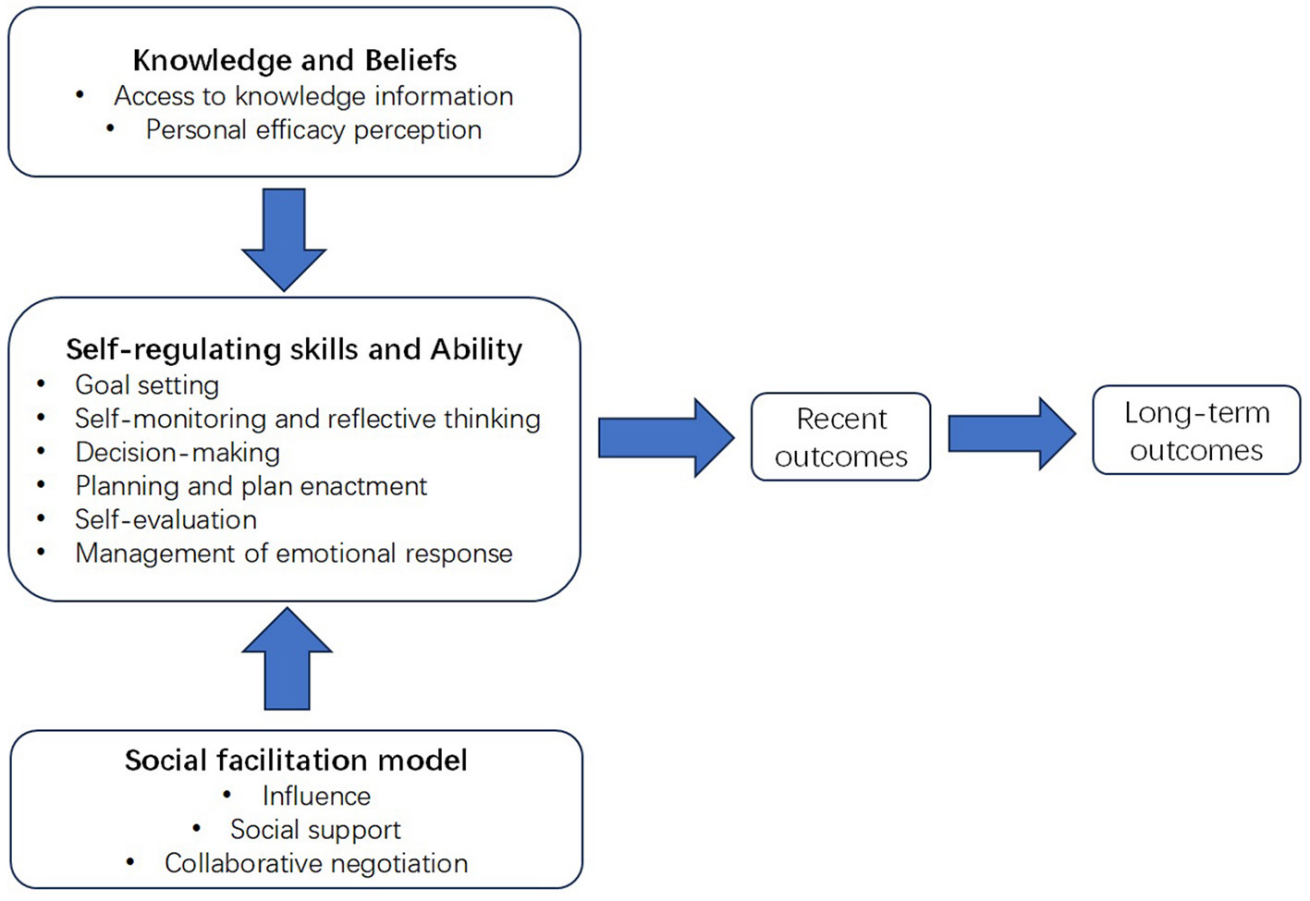
Conceptual framework of the Integrated Theory of Health Behavior Change (ITHBC). The figure depicts the three core constructs of the ITHBC—knowledge and beliefs, self-regulation skills and abilities, and social facilitation—and their interrelationships. These constructs form the theoretical foundation for this study and provide a conceptual basis for understanding health behavior change among esophageal cancer patients. The framework informs the overall direction of intervention development without depicting the specific functional modules of the mini-program.

The mini-program's functional modules are initially defined through multidisciplinary team discussions, a review of existing literature, and alignment with the ITHBC framework (Figure 2). To provide a preliminary overview of the planned intervention components, a draft list of core functional modules is presented in Table 3. These modules are subject to further refinement based on findings from qualitative research and expert panel consultation, to ensure responsiveness to patient needs. For example, if psychological support is found to be of lower relative priority based on patient feedback, the corresponding module may be adjusted or integrated into broader support features. In addition, expert consensus will also evaluate the feasibility and prioritization of each module, strengthening the intervention's scientific foundation and practical value.

**Table 3:** Preliminary functional components of the digital health intervention based on ITHBC.

**Functional module**	**Tentative description**	**Corresponding theoretical construct (ITHBC)**
Personal Center (“Me”)	User profile, disease stage information, feedback, privacy settings, data archive	Self-monitoring, Engagement
Symptom Tracker	Daily symptom logging, warning alerts, and visualized reports	Self-regulation, Self-monitoring
Knowledge Hub	Provides curated Q&A-style health education tailored to different disease stages	Knowledge & Belief
Health Reminders	Covers medication, appointments, and physical activity reminders	Self-regulation Skills
Emotional Support	Delivers coping strategies or access to psychological resources	Social Facilitation, Emotional Support
Peer and Professional Support	Facilitates peer interaction, expert Q&A, and experience sharing	Social Facilitation, Belief Support
Diet and Nutrition	Offers adaptive dietary guidance and interactive nutrition tools	Knowledge & Belief, Self-regulation Skills

### 2.3 Phase 3: evaluation

Upon completion of development, the mini-program will undergo a mixed-method usability evaluation. Quantitative measures will draw on the mHealth App Usability Questionnaire (MAUQ), demographic and eHealth literacy (eHEALS) questionnaires, backend interaction analytics, and structured task-based performance metrics. To complement these data, brief post-test interviews will be conducted to explore users' experiences, perceived barriers, and improvement suggestions. Insights derived from integrating performance metrics with user narratives will guide iterative refinements and ensure that the program meets the needs of patients, caregivers, and healthcare professionals. All usability testing procedures and outcomes will be documented in accordance with ISO/IEC 25062 (49) (Common Industry Format for Usability Test Reports) for standardized and comprehensive reporting.

#### 2.3.1 Sample/participants

A convenience sampling method with consecutive recruitment will be used to enroll three participant groups: esophageal cancer patients, their primary caregivers, and healthcare professionals. The expected sample size for each group will be described in detail in subsequent sections. Eligibility criteria include age ≥18 years, provision of written informed consent, and basic smartphone literacy.

All participants will continue to receive routine clinical care and follow-up as prescribed by their healthcare providers. The use of the digital health intervention will not interfere with any ongoing medical treatment or supportive care. No specific concomitant interventions are restricted or prohibited during the study.

**(1) Patients**

Inclusion criteria: histologically confirmed esophageal cancer at the diagnostic, treatment, or recovery phase. ability to independently perform basic smartphone operations.

Exclusion criteria: severe cognitive impairment (Mini-Mental State Examination [MMSE] score <24) or psychiatric disorders. coexisting advanced malignancies or terminal diseases with an expected survival of <6 months. inability to understand Chinese or complete the testing procedures.

**(2) Primary caregivers**

Inclusion criteria: directly providing care for the patient for ≥ 20 h per week for ≥1 month. no severe visual or hearing impairments.

Exclusion criteria: presence of severe chronic illnesses. conflicts of interest with the patient (e.g., legal disputes).

**(3) Healthcare professionals**

Inclusion criteria: oncologists, thoracic surgeons, or nurses with ≥2 years of clinical experience in EC care. Having managed ≥ 5 EC patients in the past 6 months.

Exclusion criteria: involvement in the development of similar digital tools within the past year. Inability to complete the full usability testing process.

#### 2.3.2 Sample size and recruitment

A stratified sampling strategy will be employed to ensure representation of key user groups and disease phases. Evidence from usability research indicates that approximately 10–12 participants are sufficient to identify the majority of usability issues (50, 51). To obtain sufficiently stable descriptive metrics, the planned sample sizes are set as follows: 12–18 patients across major disease phases, 8–12 primary caregivers, and 8–12 healthcare providers. Allowing for an anticipated 20% attrition rate, the target sample size is 34–40 participants. Participants will be recruited through ward posters, outpatient clinic flyers, and referrals from healthcare staff. Eligible individuals will be identified, provided with detailed study information, and enrolled upon obtaining written informed consent. Qualitative feedback will be collected from the same participants through brief post-test interviews. As these interviews are intended to inform formative usability evaluation and identify improvement needs, no formal sample size calculation is required for this component.

#### 2.3.3 Data collection and management

Prior to formal testing, all participants will receive a system operation manual and familiarize themselves with the mini-program's interface and functions. To simulate real-world usage, no additional structured training will be provided. Participants will engage with the mini-program for 1 month, with technical support available upon request.

Data will be collected at two time points: baseline (T0, enrollment) and follow-up (T1, 1 month after initial use). At T0, demographic characteristics will be recorded, together with the validated 8-item eHealth Literacy Scale (eHEALS) (52). At T1, online surveys will be distributed via the Wenjuanxing platform (Version 3.0.10) to assess system usability (MAUQ) and self-reported task performance. Meanwhile, user interaction data will be automatically collected through the mini-program's backend, including function usage frequency, session duration, and time spent on each page. These metrics will be used to characterize actual engagement behaviors and interaction patterns. A purposive subsample of participants will also be invited to complete brief semi-structured interviews at T1. Interviews will be audio-recorded, transcribed verbatim, and organized for qualitative analysis.

All data will be independently entered and cross-checked by two researchers to ensure accuracy. Data collection and management will adhere to a predefined protocol, with personally identifiable information anonymized during storage and analysis. Access to data will be restricted to authorized research personnel.

#### 2.3.4 Outcomes measures

**(1) Primary outcome**

- mHealth App Usability Questionnaire

The usability of the mini-program will be assessed using the Chinese version of the mHealth App Usability Questionnaire (MAUQ). The MAUQ comprises 21 items across three dimensions: ease of use and satisfaction, system information arrangement, and usefulness. Each item is rated on a 7-point Likert scale (1 = strongly agree; 7 = strongly disagree). The overall usability score is calculated as the average of all item responses, with scores closer to 1 indicating better usability. The Chinese version has demonstrated good reliability and validity, with a total questionnaire Cronbach's α of 0.912 and dimension-specific Cronbach's α values of 0.912, 0.884, and 0.907, respectively (53).

**(2) Secondary outcomes**

- Task performance

As part of the usability evaluation, task performance will be assessed to objectively measure users' ability to navigate and interact with the mini-program. A set of representative tasks will be developed based on the system's core functionalities, simulating common usage scenarios. The evaluation will be conducted in accordance with the usability principles outlined in ISO 9241–11 (54). At the end of the trial, participants will be asked to independently complete these predefined tasks. The Task Completion Rate (TCR)—defined as the percentage of users who successfully complete the assigned tasks—will serve as a key usability metric. A TCR of ≥80% is generally considered indicative of good usability (55), though this threshold may vary depending on task complexity and context. Additionally, Task Completion Time (TCT) will be recorded to assess task efficiency, while the Error Rate (ER) will quantify operational inaccuracies. The Assistance Rate (AR)—indicating the level of external support needed—will be used to evaluate users' reliance on external guidance.

**(3) Other measurements**

- General information questionnaire

Demographic and clinical data will be collected using a custom-designed questionnaire developed by the research team. Demographic variables include age, sex, residence, employment status, education level, marital status, family monthly income per capita, primary caregiver, and medical expense payment method. Clinical variables include histological type of EC, tumor staging, and pathological classification.

- eHealth literacy

eHealth literacy will be measured using the 8-item eHealth Literacy Scale (eHEALS), which evaluates individuals' perceived ability to seek, understand, and use electronic health information. Each item is rated on a 5-point Likert scale, with higher scores indicating better eHealth literacy. The eHEALS has been widely validated across diverse populations and consistently demonstrates good internal consistency and construct validity (56).

#### 2.3.5 Statistical analysis

Data will be analyzed using IBM SPSS Statistics version 29.0 (IBM Corp.). The primary outcome, overall usability measured by the Chinese version of the mHealth App Usability Questionnaire (MAUQ), will be summarized using mean ± standard deviation (SD) and 95% confidence intervals (CI).

For secondary outcomes, the task completion rate (TCR, %) will be evaluated using a one-sample proportion test to assess whether it reaches the predefined usability benchmark. Other task performance metrics, including task completion time (TCT), error rate (ER), and assistance rate (AR), will be analyzed according to normality test results. Normally distributed variables will be presented as mean ± SD, and non-normally distributed variables as median with interquartile range (IQR).

Demographic and clinical characteristics, along with eHEALS scores, will be summarized using descriptive statistics. Categorical variables will be reported as frequencies and percentages [*n* (%)], while continuous variables will be presented as mean ± SD or median (IQR), depending on the data distribution.

Qualitative data from post-test interviews will be analyzed using conventional content analysis. Two researchers will independently code the transcripts, compare and reconcile coding discrepancies through discussion, and develop categories and themes following established analytic procedures. Qualitative findings will be integrated with quantitative usability outcomes to support a comprehensive interpretation of overall usability.

## 3 Discussion

The presented study protocol outlines a patient-centered WeChat-based digital health intervention for esophageal cancer patients, developed through Patient Journey Mapping (PJM) and informed by the Integrated Theory of Health Behavior Change (ITHBC).

Unlike previous mHealth interventions for cancer patients that often lack user engagement and contextual relevance (29, 57), this study incorporates PJM to systematically identify health management needs across the care continuum. This user-informed approach improves alignment with patient experiences and addresses critical gaps in existing care pathways, which may inform future refinement of digital health strategies and clinical services.

Furthermore, the mini-program has the potential to enhance patient self-management and optimize healthcare resource utilization. At the individual level, a disease-specific digital health tool may improve patients' disease awareness, strengthen self-management capabilities, and ultimately enhance their quality of life (58). Compared to previous mHealth studies, this program offers advantages in interactivity, allowing for a more personalized and adaptive user experience. At the system level, it could help reduce avoidable hospital visits and streamline care delivery, thereby potentially lowering medical costs.

Third, the mixed-method usability evaluation enhances the interpretability of findings. Quantitative metrics provide objective evidence of system performance, whereas post-test interviews help uncover contextual factors and latent user needs that cannot be captured by metrics alone. This integrated approach aligns with recommended practices for early-stage digital health evaluation and supports targeted, evidence-informed refinement of the mini-program.

This study integrates PJM and ITHBC to inform the development of a targeted digital health intervention. By grounding the design in patient needs and behavioral theory, the mini-program is expected to promote user engagement and adherence. Although tailored to EC patients, this methodological approach may provide a useful reference for developing digital health tools for other chronic disease populations.

## 4 Limitations

This study has several limitations that should be acknowledged. First, the sample is drawn from a single center, which may limit the generalizability of findings to other geographic or clinical settings. Second, variations in digital literacy, particularly among older adults, may limit full engagement with the mini-program. Although eHealth literacy will be measured using the eHEALS, individuals with very low digital skills may remain under-represented. Third, the study primarily focuses on early-stage usability evaluation rather than clinical effectiveness. Future evaluations, once the prototype is stabilized, may adopt more rigorous comparative designs (e.g., randomized or cross-over trials where appropriate) and assess clinical endpoints such as quality of life, symptom control, and health-management behaviors to evaluate effectiveness and support potential scalability.

## 5 Conclusion

This study develops and evaluates a WeChat-based mini-program to support personalized health management for patients with EC. The anticipated findings may enrich the evidence base for digital health interventions and offer practical insights for optimizing and scaling mHealth tools. While this study primarily focuses on usability over clinical efficacy, its methodological framework and multidimensional evaluation approach lay the groundwork for further research. Future studies should assess its impact on self-management, healthcare resource utilization, and health outcomes, as well as explore its integration and scalability in clinical practice.

## Data Availability

This manuscript reports a study protocol; no data have been collected or analyzed at this stage. In accordance with the FAIR Guiding Principles (59), any data generated during the study will be securely stored on institutional servers and made findable and accessible to authorized researchers upon reasonable request. Data will be documented in a standardized format to ensure interoperability and enable reuse in future research, while maintaining participant confidentiality and ethical considerations.
